# History of a Case of a Wound Penetrating the Thoracic Cavity, with Some Considerations on the Causes of Emphysema

**Published:** 1820-12

**Authors:** Baron Larrey


FOR THE LONDON MEDICAL AND PHYSICAL JOURNAL.

*History of a Case of a Wound penetrating the Thoracic Cavity, with some Considerations on the Causes of Emphysema.* By Baron Larrey.

I HAVE had occasion to observe, in several articles in my *Campagnes Chirurgicales,* that all wounds penetrating the thoracic cavity, made by a cutting weapon, were generally followed by more or less extensive emphysema: it is, indeed, difficult to conceive that a pointed and cutting instrument could penetrate one of the thoracic cavities to a certain depth without wounding the lung, which exactly fills that cavity, and without a degree of emphysema proportionate to the extent of the lesion, and to the want of parallelism between the intercostal opening and that of the integuments. There may, however, be wounds penetrating the thorax, with laceration of the parenchymatous structure of the lungs and expectoration of blood, without either external emphysema or collection of fluids in the thoracic cavity: it is when the point of the weapon which made the wound is not very sharp, as, for example, that of a common fencing-foil. The serous tunic of the lungs may indeed not be divided,—giving way, by means of its density and elasticity, to the impulse of the blunt point of the weapon,—whilst the subjacent pulmonary vessels are contused and lacerated to some depth: whence emission of blood, the effusion of which takes place by the bronchiæ, larynx, and mouth, and not at all by the intercostal wound, which does not either give vent to air; and collection of blood in the chest takes place only when the intercostal vessels are also wounded.

Each of those various conditions presents its respective signs. Thus, when the instrument, after having pierced the thorax and penetrated to a certain depth, has not divided the pleura of the lung, although this organ has been struck by the instrument, there may ensue, and undoubtedly there will ensue, expectoration of blood in more or less abundance, without any trace of emphysema externally; and, when the intercostal artery has not been cut or lacerated, which happens but rarely, the patient will be free from any collection of blood in the chest. We have here to fear only the consequences dependant on irritation and inflammation; which are to be prevented by a free extension, by incision, of the external wound, and appropriate dressings to it; by the application of depletory and revulsive cuppingglasses, (*i. e.* with and without the scarificator;) by laxative and mucilaginous drinks, and general blood-letting if it is indicated.

The following case will show more clearly the nature of this sort of injury, and the phenomena which accompany it.

M. J. Elbin, a fusileer in the second regiment of the Infantry Guards, twenty-three years of age, was accidentally wounded, in the right side of the chest, with the point of a foil, by one of his comrades, whilst fencing with him. After having perforated the pectoral muscle, above the right nipple, the instrument penetrated the cavity of the chest, between the third and fourth ribs, to the depth of about an inch and a half. Elbin instantly fell: he lost but little blood by the wound, but he immediately expectorated a large quantity. He was removed, a few minutes afterwards, to the hospital *Gros Caillou.* The surgeon on duty administered the aid immediately requisite, and the next day, on my visit, I found him in the following state: A small wound, of a square figure, occupied the point of the chest above mentioned; it hardly permitted the passage of a flexible probe into this cavity: the pulse was small, accelerated, and feeble; the face was pale; the extremities were cold. The patient breathed with difficulty; he complained of acute pains in the wounded parts, and that the slightest movements added to his sufferings. There was not the least sign of emphysema apparent externally, not even around the wound; yet the hemoptysis, although less abundant, continued to exist.

I extended freely the external wound, and laid bare the intercostal muscles, after having applied a dry cupping-glass over the incision, which produced a complete disgorgement of the surrounding cellular tissue, and brought from the cavity of the chest a large quantity of thick black blood. I then brought together the edges of the wound; fixed them in contact by means of two small stripes of adhesive-plaster, over which a latticed piece of linen, spread with ointment of styrax, soft pledgets, compresses, and a bandage, were applied. The patient now felt much relieved: he breathed more freely, and greater freedom of the circulation was promptly re-established. Some febrile commotion, with signs of local irritation, came on in the course of the same evening, which was appeased, and its consequences prevented, by blood-letting from the arm; the successive application, on the right side and around the wound, of dry cupping-glasses guarded by a fold of linen; the internal use of iced, acidulated, and mucilaginous, drinks; and by chicken-broth, as the only nutriment. The symptoms gradually subsided; the patient continued to improve, and soon became convalescent.

On the fifteenth day from the occurrence of the injury, a vesicatory was applied to the whole of the right side of the chest, which perfected the cure. The patient left the hospital, to resume his duty, thirty-five days after his admission.

